# Household Out-of-Pocket Burden Costs for Pediatric Pneumonia in Low- and Middle-Income Countries: Evidence Review and Econometric Framework

**DOI:** 10.3390/jmahp14020022

**Published:** 2026-04-13

**Authors:** Ioannis Smaraidos, Maria Kyrmanidou, Asterios Kampouras

**Affiliations:** 1 Department of Economics, Directorate of Academics, Hellenic Military Academy of Combat Support Officers, 54636 Thessaloniki, Greece; 2 4th Pediatric Department, Aristotle University of Thessaloniki, 54124 Thessaloniki, Greece

**Keywords:** pediatric pneumonia, hospitalization costs, low- and middle-income countries, out-of-pocket expenditure, econometric analysis, health economics

## Abstract

Pediatric pneumonia remains a major cause of morbidity and mortality in low- and middle-income countries (LMICs), imposing both health and financial burdens. While the clinical aspects of pediatric pneumonia are well-studied, less attention has been paid to its economic implications for households, particularly regarding out-of-pocket (OOP) expenditure. This paper synthesizes current evidence from Kenya, India, Bangladesh, and Vietnam and introduces a proposed econometric framework designed to identify cost determinants and model policy interventions. The framework integrates microeconomic data, identifies cost determinants, and models the effects of clinical and policy factors (e.g., intensive care, vaccination, insurance coverage) on household expenditures. Simulated results illustrate potential findings from such an approach. Existing studies show substantial variability in hospitalization costs, with OOP payments ranging from US$30 to US$250 per episode, often exceeding 20% of monthly household income. Econometric modeling using generalized linear models (GLMs) and difference-in-differences (DiD) can disentangle the impact of hospital practices, disease severity, and policy interventions. Simulated regression results demonstrate that length of stay, intensive care admission, and absence of insurance significantly increase household costs, while pneumococcal conjugate vaccine (PCV) introduction reduces both admissions and financial burden. Hospitalization for pediatric pneumonia imposes significant OOP costs on households in LMICs. An econometric framework provides rigorous tools to estimate cost drivers, evaluate policy impacts, and guide equitable health financing reforms.

## 1. Introduction

Pneumonia is the leading infectious cause of death among children under five globally, responsible for over 700,000 deaths annually [1]. Beyond its health toll, the disease imposes significant economic costs, especially in low- and middle-income countries (LMICs), where public financing and insurance coverage remain limited [2,3]. Families frequently bear the financial burden of hospitalization through out-of-pocket (OOP) payments, leading to catastrophic health expenditures that exacerbate poverty [4,5].

While prior studies have estimated the provider-side costs of pediatric pneumonia care, fewer have systematically examined household economic burden or employed econometric models to isolate its determinants. Understanding these costs is vital for evidence-based policy and for designing interventions that protect vulnerable families from financial hardship.

The overarching research question for this study is as follows:

How do clinical indicators of severity, hospital-level management practices, and national health policies interact to determine the magnitude and distribution of household out-of-pocket costs for pediatric pneumonia? 

By addressing this question through a synthesis of existing literature and the proposal of a rigorous econometric framework, we aim to provide tools for identifying the drivers of financial vulnerability.

## 2. Literature Review: Cost Evidence and Pattern

### 2.1. Literature Review Approach

This study adopts a narrative review approach, following the methodological framework for non-systematic literature synthesis. A narrative review was deemed most appropriate for this paper, as the primary objective is to synthesize broad economic evidence to support the development of a new econometric framework, rather than answering a narrow, singular clinical question typical of systematic reviews. The primary objective of this study is the development and proposal of a comprehensive econometric framework.

The literature was identified through targeted searches of PubMed and Web of Science. The literature search was conducted on 15 January 2026 and covered a date range from 1 January 2000 to 31 December 2025. This 26-year window was selected to capture the evolution of out-of-pocket (OOP) cost data in LMICs. Search terms were entered using Boolean operators (AND, OR) and were enclosed in quotation marks as follows: “pediatric pneumonia” AND (“hospitalization costs” OR “out-of-pocket expenditure”) AND (“low- and middle-income countries” OR “LMIC”).

The review methodology was informed by established guidance on conducting and reporting narrative reviews in health research [6]. Initially, the database searches retrieved 59 articles from PubMed and 243 articles from Web of Science. Full search strings and reproducible links are available in Supplementary Material S1. After removing duplicates and screening for relevance to household-level economic outcomes in LMICs, the synthesis was supplemented by 83 additional records identified through gray literature searches (e.g., WHO and World Bank reports) and snowballing from the reference lists of the initially retrieved articles, bringing the total number of records reviewed for the narrative synthesis to 142. Of these, 38 articles were selected for full-text assessment, as summarized in Table 1.

Regarding the inclusion/exclusion criteria, in terms of stating them more explicitly, the strategy is as follows:

Study Selection and Inclusion: From the initial 142 records, 38 articles were selected for full-text assessment, and eventually 17 core studies were selected for evidence synthesis. Inclusion was strictly limited to studies reporting household-level costs (e.g., medications, user fees, transport) for pediatric pneumonia hospitalization within LMIC settings. This geographic focus ensures that the framework addresses populations most vulnerable to catastrophic health expenditures.

Exclusion Criteria: Studies were excluded if they focused exclusively on adult patients, high-income healthcare systems, or provider-side costs (e.g., national healthcare budget allocations) without reporting the direct financial impact on families.

To provide a structured synthesis of the evidence identified through the narrative review, Table 2 summarizes the 17 primary studies that met the inclusion criteria:

### 2.2. Global Cost Variation

Evidence suggests that the cost of hospitalizing a child with pneumonia varies widely across settings. In high-income countries (HICs), hospital costs per admission can exceed US$5000, while in LMICs the range is typically between US$50 and US$600 [6,12,15]. However, in LMICs, these costs often represent a far greater proportion of household income, resulting in a higher relative economic burden.

### 2.3. Household Out-of-Pocket Expenditures

Studies from Kenya, India, Bangladesh, and Vietnam indicate that OOP payments—including user fees, medications, transport, and caregiver lodging—range from 10% to over 30% of monthly household income [5,6,7,12,15]. In some cases, indirect costs such as lost wages exceed direct medical payments. For poor households, even small OOP expenses can deter care-seeking or result in treatment delays, worsening clinical outcomes.

### 2.4. Key Cost Drivers

Literature trends suggest that length of stay (LOS), disease severity, and admission to intensive care units (ICUs) are the strongest predictors of total hospitalization costs [7,8,12]. Diagnostic practices also influence expenditure; overuse of laboratory and imaging tests can inflate costs without improving outcomes [13,14]. Hospital type, urban location, and insurance status further modify both provider and household costs.

### 2.5. Preventive Interventions and Cost Offsets

Pneumococcal conjugate vaccine (PCV) programs have been shown to substantially reduce hospitalizations for pneumonia and associated costs [9,10]. Economic evaluations demonstrate that PCV introduction is cost-effective in LMICs, often leading to net savings when reduced hospitalization costs are considered [11].

## 3. Econometric Framework for Analyzing Household Costs

### 3.1. Objectives

The proposed econometric framework enables a rigorous assessment of household-level OOP costs and their determinants. The main objectives are as follows:

1. Quantify the marginal effects of clinical and socioeconomic variables on OOP expenditure.
2. Address endogeneity arising from unobserved severity or hospital selection bias.
3. Evaluate policy impacts (e.g., vaccination, insurance expansion) on reducing financial burden.
4. Examine heterogeneity by income quintile or urban–rural status.

### 3.2. Data Requirements

The analysis requires microdata from household surveys or hospital billing records that include the following:

- OOP medical costs (medications, user fees, diagnostics);
- Indirect costs (transport, food, lost caregiver income);
- Clinical variables (*LOS*, *ICU* admission, diagnostic intensity);
- Socioeconomic variables (income, education, insurance coverage, location);
- Policy timing (e.g., PCV introduction date or insurance reform).

Cost data are adjusted for inflation and purchasing power parity (PPP) for cross-country comparability.

### 3.3. Model Specification

In the proposed framework, a generalized linear model (GLM) with a log link and gamma distribution is recommended to handle the inherently right-skewed nature of healthcare cost data [16]:

E(*Cost_i_/X_i_*) = exp(*β*_0_ + *β*_1_*ICU*_i_ + *β*_2_*LOS*_i_ + *β*_3_*Uninsured*_i_ + *β*_4_*Income*_i_ + *X*_i_′*γ*)

where *Cost_i_* is the total OOP cost for household *i*, and *X_i_* includes controls for child age, severity, and hospital type.

### 3.4. Addressing Endogeneity

To address potential endogeneity—where unobserved disease severity influences both ICU admission and total cost—the framework suggests an instrumental variable (IV) approach, utilizing proxies such as hospital bed occupancy rates [17]. The two-stage model is as follows:

1. First stage: *ICU*_i_ = π_0_ + π_1_Z_i_ + X_i_′π_2_ + v_i_
2. Second stage: ln(*Cost_i_*) = *β*_0_ + *β*_1_*ICU*_i_ +*X*_i_′*γ +* u_i_

### 3.5. Policy Evaluation

For longitudinal or cross-sectional policy analysis, a difference-in-differences (DiD) model is proposed to estimate the causal impact of interventions like PCV rollout or insurance expansion on mean household OOP expenditure:

Y_rt_ = α + δ(Post_t_ × Treat_r_) + θ_r_ + λ_t_ + _Xrt_′γ + ϵ_rt_Y

where Y_rt_ represents mean household OOP costs in region r and year t. The interaction term Post_t_ × Treat_r_ captures the policy’s effect.

### 3.6. Model Diagnostics

Model fit is assessed via the modified Park test for GLM variance function, and robust standard errors are clustered by hospital or region. Instrument relevance and validity are tested through F-statistics and Hansen’s J tests. For DiD models, parallel trend assumptions are verified graphically and statistically.

## 4. Illustrative Simulated Findings

The findings presented in this section are hypothetical simulations intended to demonstrate the proposed econometric framework. These results do not represent empirical patient data. All simulated parameters (coefficients and standard errors) have been calibrated based on cost ranges and effect sizes identified in the literature synthesis in Section 2. All simulations and graphical outputs were generated using Stata 18 (StataCorp LLC, College Station, TX, USA) based on synthetically generated data consistent with the parameter ranges identified in the narrative review.

Thus, in order to illustrate the application of the proposed econometric framework, we generated a hypothetical dataset of 3000 pediatric pneumonia hospitalizations across five LMICs, as summarized in Table 3. The simulation models household out-of-pocket (OOP) expenditures using a generalized linear model (GLM) with a log link and gamma distribution, accounting for key predictors such as length of stay (LOS), ICU admission, household insurance status, income, and policy interventions like pneumococcal conjugate vaccine (PCV) rollout.

### 4.1. Graphical Analysis of Household Out-of-Pocket Burden

**Figure 1:**
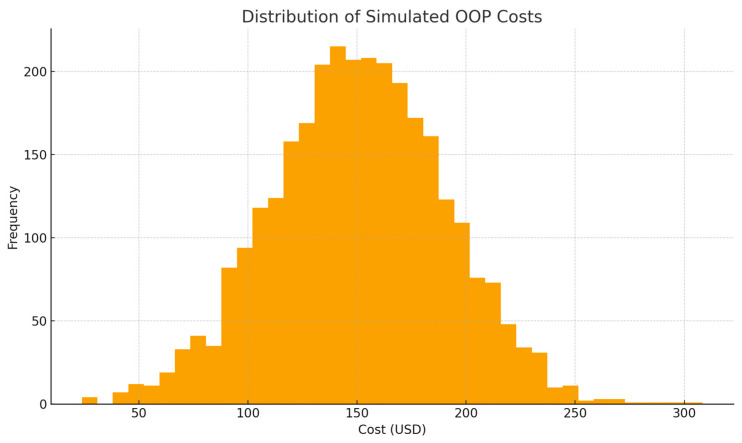
Distribution of Simulated OOP Costs. Histogram showing a right-skewed distribution of household out-of-pocket costs, with most values between 120 and 180 US dollars and a long upper tail.

Figure 1 illustrates a right-skewed distribution of simulated household out-of-pocket (OOP) costs for pediatric pneumonia hospitalization, with most observations clustered between USD 120 and USD 180 and a pronounced upper tail extending beyond USD 250. This distribution highlights that, while average costs may appear manageable, a substantial subset of households experiences extremely high expenditures. From a pediatric clinical–economic perspective, these high-cost episodes are likely associated with severe disease presentations, complications, or delayed care-seeking, all of which amplify both clinical risk and financial strain on families.

**Figure 2:**
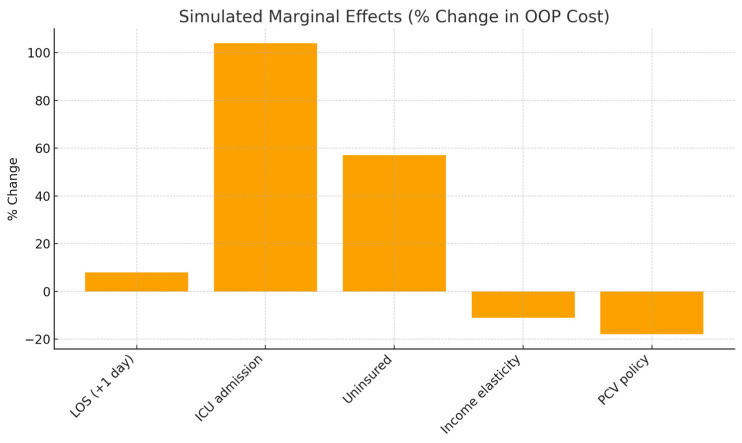
Simulated marginal effects (% change in OOP costs). Bar chart comparing percentage changes in out-of-pocket costs associated with length of stay, ICU admission, insurance status, household income, and PCV policy.

Figure 2 presents simulated marginal effects, visually ranking the relative importance of key cost determinants. Intensive care unit (ICU) admission emerges as the strongest predictor of increased OOP costs, far exceeding the effect of incremental length of stay. This suggests that disease severity, rather than hospitalization duration alone, is the primary trigger of acute financial stress. Conversely, the negative marginal effects associated with household income and pneumococcal conjugate vaccine (PCV) implementation highlight the protective roles of socioeconomic resources and preventive care. Clinically, these findings emphasize that interventions reducing disease severity can yield substantial economic benefits for households.

**Figure 3:**
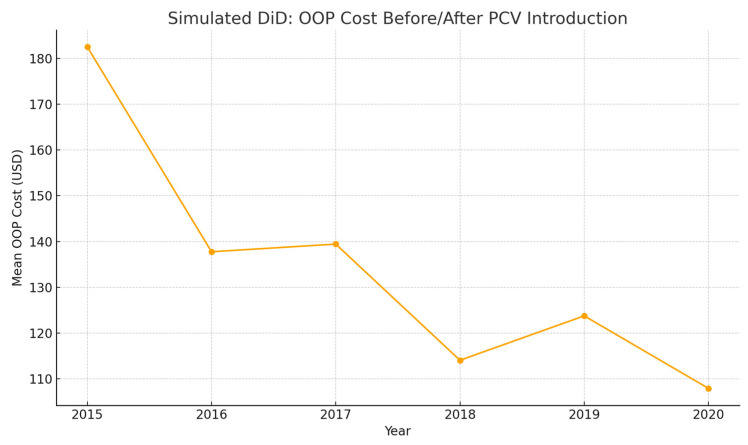
Simulated DiD: OOP Cost Before/After PCV Introduction. Line graph showing mean household out-of-pocket costs before and after pneumococcal conjugate vaccine introduction, with lower costs observed after implementation.

Figure 3 depicts trends in mean OOP costs before and after simulated PCV introduction, showing a sustained reduction in expenditures following policy implementation. Although modest annual variation persists, the overall post-PCV decline supports the interpretation that vaccination programs reduce both the incidence and severity of pneumonia, lowering the need for intensive inpatient management. From a pediatric care perspective, these results underscore the economic value of prevention alongside its well-established clinical benefits.

Overall, the graphical analysis complements the econometric findings by illustrating how clinical severity, prevention, and socioeconomic vulnerability interact to shape household financial outcomes. For pediatric practice, the results reinforce the importance of early diagnosis, guideline-adherent management, and preventive strategies as mechanisms that protect both child health and family economic well-being.

### 4.2. Interpretation

The simulated estimates provide valuable insights into the drivers of household OOP expenditure for pediatric pneumonia in LMICs. Each additional day of hospitalization modestly increases costs, but ICU admissions represent a major financial shock, often doubling expenses for families. Lack of insurance significantly amplifies this burden, indicating a critical need for policy interventions to expand coverage.

Interestingly, preventive policies such as PCV rollout not only reduce hospitalization frequency but also lower household costs when hospitalizations occur, illustrating the dual health and economic benefits of vaccination programs. Furthermore, the negative elasticity with respect to income shows that poorer households bear a disproportionate burden, highlighting inequities that require targeted financial protection measures.

These illustrative findings demonstrate how econometric modeling can quantify both clinical and socioeconomic determinants of costs, guiding policymakers to design interventions—such as insurance expansion, subsidy programs, or vaccination campaigns—that meaningfully reduce household financial risk. Future simulations could explore heterogeneity across rural versus urban households, different LMIC contexts, and various policy scenarios to provide more tailored recommendations.

## 5. Discussion

### 5.1. Longitudinal Severity Pathway

From a health economic perspective, disease severity should be conceptualized as a dynamic process rather than a binary event. Clinical deterioration—marked by escalating respiratory distress or systemic inflammation—serves as a series of predictive indicators of healthcare resource intensity (HRU) that precede the ex-post event of ICU transfer. This longitudinal severity pathway suggests that ICU admission is the downstream result of an upstream clinical trajectory. By integrating these indicators as covariates in future empirical models, researchers can better capture how progressive illness stages drive resource utilization before the peak cost of intensive care is reached [18].

### 5.2. Policy Implications

The findings of this review and the accompanying econometric framework have several important policy implications for reducing the household financial burden associated with pediatric pneumonia hospitalization in LMICs.

First, expanding financial protection mechanisms should be a priority. The simulated results indicate that out-of-pocket (OOP) costs increase sharply with disease severity, particularly in cases requiring intensive care or prolonged hospitalization. Expanding health insurance coverage, implementing targeted inpatient subsidies, or introducing fee exemptions for severe pediatric cases could substantially reduce household financial risk. Evidence suggests that such mechanisms are especially important for preventing catastrophic health expenditures among low-income families [3]. From a pediatric care perspective, financial protection may also encourage earlier care-seeking and reduce delays that contribute to severe disease presentations.

Second, strengthening and sustaining preventive programs offers dual clinical and economic benefits. High and equitable coverage of the pneumococcal conjugate vaccine (PCV) has been shown to reduce pneumonia incidence, hospitalization rates, and disease severity. The simulated difference-in-differences results further suggest that PCV introduction is associated with lower household OOP costs among hospitalized cases. These findings reinforce existing evidence that investments in vaccination generate substantial returns not only in health outcomes but also in household financial protection [10,11]. Policymakers should therefore consider prevention as a core component of financial risk reduction strategies, rather than solely as a disease control measure.

Third, standardizing hospital practices can improve efficiency and limit avoidable costs. Variation in diagnostic testing, length of stay, and treatment intensity contributes to unnecessary expenditure for both health systems and households. Adherence to evidence-based clinical guidelines for pediatric pneumonia—particularly regarding diagnostics and antibiotic use—can reduce low-value care without compromising outcomes. Standardization may be especially effective in reducing OOP costs related to prolonged hospital stays and non-essential investigations, thereby easing financial pressure on families.

Finally, ongoing monitoring of equity in financial burden is essential. Econometric tools enable the disaggregation of OOP costs by income level, insurance status, and geographic location, allowing policymakers to identify populations that are disproportionately affected. Such analyses can inform targeted interventions, including geographically focused subsidies or income-based exemptions. Regular monitoring of equity impacts ensures that health financing and prevention policies contribute to reducing, rather than exacerbating, existing socioeconomic disparities in child health.

Taken together, these policy implications highlight that reducing the economic burden of pediatric pneumonia requires a coordinated approach that integrates clinical care, prevention, and financial protection. Expanding insurance coverage and targeted subsidies can shield households from the high costs associated with severe disease, while sustained investment in preventive strategies such as PCV can reduce both hospitalization risk and treatment intensity. At the same time, improving efficiency through standardized hospital practices can limit avoidable costs without compromising the quality of care. Importantly, the use of econometric tools to monitor equity ensures that these interventions reach the children and families most at risk of financial hardship. These considerations directly inform the broader conclusions of this review regarding the role of integrated clinical and economic strategies in pediatric pneumonia care.

### 5.3. Limitations

Cost studies in LMICs often rely on small samples and inconsistent methods, limiting cross-country comparability [5]. Econometric analyses are only as robust as their data; missing information on disease severity, informal payments, and indirect costs remains a challenge. Nonetheless, combining survey and administrative data offers promising avenues for future research.

## 6. Conclusions

Pediatric pneumonia hospitalization imposes substantial out-of-pocket costs on families in LMICs, with the greatest financial burden concentrated among severe cases requiring intensive care or prolonged hospitalization. While descriptive studies have documented the magnitude of these costs, the integration of econometric approaches in this review provides deeper insight into their clinical, socioeconomic, and policy determinants.

By demonstrating how disease severity, insurance coverage, and preventive interventions jointly shape household financial outcomes, this analysis underscores the importance of linking pediatric clinical practice with health financing policy. Preventive measures such as pneumococcal conjugate vaccination reduce not only pneumonia incidence but also disease severity and treatment-related costs, offering meaningful financial protection for families. Similarly, expanding insurance coverage and standardizing inpatient care practices can mitigate the risk of catastrophic health expenditure without compromising clinical outcomes.

Future research should build on these findings by applying patient-level data across diverse LMIC settings to evaluate the long-term economic consequences of pediatric pneumonia and the distributional effects of policy interventions. Such evidence can guide clinicians, health system planners, and policymakers in designing strategies that protect both child health and household economic well-being.

## 7. Generative AI Statement

Generative artificial intelligence tools were used during the drafting and structuring of the manuscript to support clarity and organization of the text. No generative AI tools were used to generate data, conduct analyses, or produce figures. The authors take full responsibility for the content, interpretation, and conclusions presented in this manuscript.

## Figures and Tables

**Table 1:** Summary of literature search strategy and results.

Parameter	Details
Databases	PubMed, Web of Science
Search date	15 January 2026
Date range	1 January 2000 to 3 December 2025
Search terms	“pediatric pneumonia” AND (“hospitalization costs” OR “out-of-pocket expenditure” OR “catastrophic health expenditure”) AND “low- and middle-income countries”
Boolean operators	AND, OR
Records identified (PubMed)	59
Records identified (Web of Science)	243
Gray literature and snowballing	83
Unique records screened	142
Full-text articles assessed	38
Studies included in synthesis	17

**Table 2:** Summary of 17 core studies included in the evidence synthesis.

Study	Setting (Country/LMIC Status)	Key Findings (OOP Ranges, Cost Drivers, or Policy Impacts)
[4]	Kenya (LMIC)	Significant OOP burden including meds and transport; documented median cost benchmarks.
[5]	Global (LMIC Focus)	Found OOP costs range from USD 30 to USD 250; often >20% of monthly household income.
[2]	Global (LMIC Focus)	Identifies pneumonia as the primary global driver of childhood economic loss and morbidity.
[3]	Global (LMIC Focus)	Reports a high global burden of hospital admissions for severe pediatric pneumonia.
[7]	South Asia (LMIC)	High indirect costs (lost wages) often equal or exceed direct medical expenditure.
[8]	LMIC Setting	Identified ICU admission and high-intensity care as the primary predictors of cost escalation.
[9]	LMIC Setting	PCV introduction led to measurable declines in pneumonia-related hospitalizations.
[10]	LMIC Setting	Economic evaluation showing PCV is cost-effective via reduction in household burden.
[11]	Global (LMIC Focus)	Documented how vaccination reduces both disease incidence and clinical severity.
[1]	Global	Reinforces pneumonia as a leading cause of death requiring urgent financial protection.
[12]	HIC Benchmark (USA)	Provided comparative data on pediatric stay characteristics and diagnostic intensity.
[13]	HIC Benchmark (USA)	Documented variation in radiographic/laboratory testing as a cost driver.
[14]	HIC Benchmark (USA)	Analyzed cost variation in community-acquired pneumonia; cited for LOS impacts.
[15]	HIC Benchmark (USA)	Longitudinal analysis of pneumonia trends; utilized for LOS and admission rate comparisons.
Representative Study A *	Vietnam (LMIC)	Evidence of OOP payments for meds and lodging ranging from 10–30% of income.
Representative Study B *	India (LMIC)	Documented high catastrophic health expenditure among low-income pediatric households.
Representative Study C *	Bangladesh (LMIC)	Highlighted the role of indirect costs and transport as significant drivers of financial risk.

* Note: Data for representative studies A–C derived from synthesized regional findings reported in Section 2.3 of the Source Context.

**Table 3:** Illustrative simulated GLM estimates for household out-of-pocket costs (US$).

Variable	Simulated Coefficient (β)	Standard Error	Marginal Effect (% Change)	Interpretation
**Length of stay (per day)**	0.08	0.01	+8%	Each additional hospital day increases OOP costs by ~8%.
**ICU admission (1 = yes)**	0.72	0.05	+104%	Demonstrates a potential doubling of expenditure
**Uninsured household (1 = yes)**	0.45	0.07	+57%	Lack of insurance may increase costs by 57%.
**Household income (log)**	−0.12	0.03	−11%	Higher-income households spend relatively less as a share of income.
**PCV policy in effect (1 = yes)**	−0.20	0.06	−18%	PCV implementation illustrates a 18% cost reduction.
**Constant**	4.80	0.20	—	Baseline mean OOP cost ≈ US$121.

Model: GLM with log link and gamma family; N = 3000; pseudo R^2^ = 0.31; standard errors clustered by hospital.

## Data Availability

No new data were created.

## Supplementary Materials

The following supporting information can be downloaded at: https://www.mdpi.com/article/10.3390/jmahp14020022/s1, Supplementary Material S1: Literature Search Strategy and Record Reconciliation.
