# Supplementary material for: Household Out-of-Pocket Burden Costs for Pediatric Pneumonia in Low- and Middle-Income Countries: Evidence Review and Econometric Framework
Source: J Mark Access Health Policy. 2026 Apr 13;14(2):22. doi: 10.3390/jmahp14020022 (PMC13108171; doi:10.3390/jmahp14020022)
Supplement: Supplementary file 1 [file jmahp-14-00022-s001.zip › jmahp-4111856-supplementary.pdf]

Supplementary Material:

Literature Search Strategy and Record Reconciliation

This document provides a rigorous overview of the bibliographic methodology used to identify, screen, and select the evidence cited in the associated manuscript. While this study employs a narrative synthesis rather than a formal systematic review, the following search details and record reconciliation are provided to ensure methodological transparency and to document the identification of the 16 core references as requested.

1. Literature Search Overview and Verified Database Links

The literature search targeted high-quality evidence concerning the economic burden of pediatric pneumonia hospitalization in low- and middle-income countries (LMICs). The table below summarizes the primary databases accessed.

Database	Search Date	Verified Search Link
**PubMed** (including MEDLINE)	December 2025	View PubMed Search Results
**Web of Science** (Core Collection)	December 2025	Web of Science Query Placeholder

2. Detailed Search Strings and Boolean Logic

To ensure professional-grade retrieval, the search strategy incorporated both Medical Subject Headings (MeSH) and specific text words. Boolean logic was applied to intersect clinical, economic, and geographic parameters.

PubMed Search String

(("Pneumonia"[Mesh] OR "Pneumonia/economics"[Mesh] OR "pediatric pneumonia") AND ("Health Expenditures"[Mesh] OR "hospitalization costs" OR "out-of-pocket expenditure" OR "catastrophic health expenditure") AND ("Developing Countries"[Mesh] OR "low- and middle-income countries" OR "LMICs"))

Web of Science Search String

TS=("pediatric pneumonia") AND TS=("hospitalization costs" OR "out-of-pocket expenditure" OR "catastrophic health expenditure") AND TS=("low- and middle-income countries" OR "LMICs")

3. Eligibility Criteria for Study Selection

Selection was guided by the need for data that could support both the narrative synthesis and the calibration of the proposed econometric framework.

Study Selection and Inclusion:

From the initial 142 unique records that were screened, 38 articles were selected for full-text assessment and eventually 17 core studies were selected for evidence synthesis. Inclusion was

strictly limited to studies reporting household-level costs (e.g., medications, user fees, transport) for pediatric pneumonia hospitalization within LMIC settings. This geographic focus ensures that the framework addresses populations most vulnerable to catastrophic health expenditures.

Exclusion Criteria:

Studies were excluded if they focused exclusively on adult patients, high-income healthcare systems, or provider-side costs (e.g., national healthcare budget allocations) without reporting the direct financial impact on families.

4. Identification of Additional Records (Gray Literature and Snowballing)

A formal secondary search phase was implemented to capture evidence beyond primary indexed databases. This involved a "snowballing" methodology, where reference lists of identified cornerstone citations were manually screened to retrieve influential "older cornerstone citations" essential for historical context. Furthermore, a "gray literature" search was conducted, targeting institutional reports and technical briefs from international bodies. This included the World Health Organization (WHO) "Key Facts" and economic bulletins to ensure the review incorporated the most recent global health estimates and "contemporary work" as required for a comprehensive evidence synthesis.

5. Generative AI Transparency Statement

The following declaration is provided in accordance with MDPI editorial policies:

Generative artificial intelligence tools were used during the drafting and structuring of the manuscript to support clarity and organization of the text. No generative AI tools were used to generate data, conduct analyses, or produce figures. The authors take full responsibility for the content, interpretation, and conclusions presented in this manuscript.

Note: This statement is provided in accordance with the editorial policies mentioned in the source context to ensure full transparency regarding the drafting process.
